# Supplementary material for: Diaper dermatitis and associated factors among children aged 0–24 months in low- and middle-income countries: A systematic review protocol
Source: PLoS One. 2025 Jan 9;20(1):e0313923. doi: 10.1371/journal.pone.0313923 (PMC11717299; doi:10.1371/journal.pone.0313923)
Supplement: S1 Checklist — (DOCX) [file pone.0313923.s001.docx]

JBI Critical Appraisal Checklist for
analytical cross sectional studies

Reviewer______________________________________ Date_______________________________

Author_______________________________________ Year_________ Record Number_________

|  | Yes | No | Unclear | Not applicable |
| --- | --- | --- | --- | --- |
| 1. Were the criteria for inclusion in the sample clearly defined? | □ | □ | □ | □ |
| 1. Were the study subjects and the setting described in detail? | □ | □ | □ | □ |
| 1. Was the exposure measured in a valid and reliable way? | □ | □ | □ | □ |
| 1. Were objective, standard criteria used for measurement of the condition? | □ | □ | □ | □ |
| 1. Were confounding factors identified? | □ | □ | □ | □ |
| 1. Were strategies to deal with confounding factors stated? | □ | □ | □ | □ |
| 1. Were the outcomes measured in a valid and reliable way? | □ | □ | □ | □ |
| 1. Was appropriate statistical analysis used? | □ | □ | □ | □ |

Overall appraisal: Include □ Exclude □ Seek further info □

Comments (Including reason for exclusion)

_______________________________________________________________________________________________________________________________________________________________________________________________________________________________________________________________________________________
