# Supplementary material for: Diaper dermatitis and associated factors among children aged 0–24 months in low- and middle-income countries: A systematic review protocol
Source: PLoS One. 2025 Jan 9;20(1):e0313923. doi: 10.1371/journal.pone.0313923 (PMC11717299; doi:10.1371/journal.pone.0313923)
Supplement: Attachment — Submitted filename: PONE-D-24-40961 Diaper Dermatitis 0-24 month LMIC .docx [file pone.0313923.s003.docx]

Protocol Number: PONE-D-24-40961

Title: Diaper dermatitis and associated factors among 0-24 months children in low- and

middle-income countries: A Systematic review protocol

I read with great interest the objectives of this data mining exercise and I am hopefully the authors will be successful. I have provided my comments that I hope will clarify what countries are in-scope and out-of-scope, what data will be analyzed, and to refine the search strategies.

My comments can be found below.

With Thanks.

Andrew Carr

**Reviewer comments:**

Overall:

It was not clear to me how “low- and middle-income countries (LMIC) will be defined. What criterion will be used?

The title of the protocol states that it will be looking at Diaper Dermatitis “and associated factors”, and the protocol states a key question as “2) What are the factors associated with diaper dermatitis among children aged 0-24 months in low- and middle-income countries?” It is not clear if this includes “causes” of diaper dermatitis, sequelae of diaper dermatitis (for example, ill temperament of the child, poor sleep, pain/crying) or other factors.

There is a statement in the “Introduction” related to the prevalence of diaper dermatitis. “Although the prevalence of DD varies from country to country, it is estimated that about 50% to 65% of infants and young children will suffer from diaper dermatitis at some time and up to 25% of children seek healthcare due to this problem (6, 7, 8).” The literature is quite scattered about the percentage of babies who experience diaper dermatitis. However, data from Visscher et al (Pediatric Dermatology Vol 17, No. 52-57, 2000) , indicates that diaper dermatitis rates are ~ 70% in the first week of life which is higher than the 50-65% reported in the publication. Further, Gustin et al (Pediatric Dermatology 2021. Jul;38(4):768-774) indicated that diaper dermatitis was found in 65-83% of newborns. Carr et al. (Pediatric Dermatology 2020 Jan;37(1):130-136) also found high rates of diaper dermatitis in Germany across 4 common sites within the diaper. Given these data, it should be noted that in reality, ‘every baby will experience diaper dermatitis as some point during their diaper wearing years’.

The Introduction also states “Its typical sign is a red, itchy, and sometimes ulcerated rash in the diaper area (perineum, peri-genital area, perianal area, and the upper thigh) occurring most often as a reaction to increased moisture due to prolonged contact with urine, feces, or retained soap or detergent (14).” I would change this to read: “It’s typical sign is a red, itchy, and sometimes ulcerated rash in the diaper area and may include the presence of papules and pustules (Buckley et al, Pediatric Dermatology Vol 33 No 6 632-639, 2016; Odio MR et al Dermatology 2000; 200:238-243). The primary diaper sites include the perineum, peri-genital area, perianal area, genital region, buttocks and groin and occur most often as a reaction to increased moisture (skin overhydration) due to prolonged contact with urine, feces, or retained soap or detergent (14).”

Introduction: “Major causes that impair the integrity of the skin are irritants in urine and feces, overhydration of the epidermal stratum and corneum, excessive skin pH, and friction against the skin (14-16).” I would reword this to say “…elevated skin pH…”

Introduction: “Disposable diapers contain absorbent chemicals that are able to absorb liquids, and have to be disposed of after usage.”. I would rephrase to state “Disposable diapers contain absorbent gel materials (AGM) that are able to absorb liquid and runny feces and are single-use.”

The Search Strategy is listed as: “The search strategy included a combination of subject terms and free text terms combined with Boolean operators (‘OR’, and ‘AND’). The Medical Subject Headings (MeSH) terms included “diaper”, OR “napkin”, OR “nappy” AND “dermatitis”, OR “rash”, OR “irritation”, AND “neonate”, OR “infant”, OR “toddler”, OR “children”, OR “pediatrics”, OR “kids”, OR “babies”, AND “developing countries”, OR “resource-limit” OR “resource-poor”, OR “low-income”, OR

“lower-middle-income”, OR “middle-upper income countries”.” I would also include “papule”, “pustule”, . I believe search terms for “income” can be included but I strongly suggest the investigators define the criterion for LMIC and then include only those publications where the study was conducted in a LMIC, as a publication may not identify its sources as LMIC.

The inclusion criteria includes data collected at the “under-five clinic”, however, given the episodic, unpredictable and short-term nature of diaper dermatitis (typically 3-10 days) the incidence of diaper dermatitis that is observed at a clinic visit may not reflect the actual rate of diaper dermatitis in the population.

The “Search date” is listed as “All research articles accessed from 20 September 2024 to 10 October 2024 without time limits will be included.”. This date is in the past.

In the exclusion criteria it states, “Studies that did not assess the magnitude of diaper dermatitis/rashes (studies that evaluate treatments for children with DD, knowledge of DD, the quality of life of children with DD, etc.) will be excluded.”. If parental habits and practices are able to be obtained, this will be critical to understand the potential causes of the diaper dermatitis, as are skin products being used, bathing frequency, diet information and family history of skin disorders, including atopic dermatitis and the like.
